# An Active Thermography approach for materials characterisation of thermal management systems for Lithium-ion batteries

**DOI:** 10.1016/j.heliyon.2024.e28587

**Published:** 2024-03-27

**Authors:** Francesca Curà, Raffaella Sesana, Luca Corsaro, Marie Marguerite Dugand

**Affiliations:** a Department of Mechanical and Aerospace Engineering, Politecnico di Torino, Corso Duca Degli Abruzzi 24, 10129, Torino, Italy; b PRODUCT DEVELOPMENT – BCI - CRF - Materials & Sustainability Engineering - South Europe Technical Center EEs & E-PWT - Optics & Glazing Team, C.so Settembrini 40, 10235, Turin, Italy

**Keywords:** Phase changes material (PCM), Thermal management system (TMS), Active thermography (AT), Thermal conductivity, Thermal diffusivity, ISO 18755 Standard, Analytical model

## Abstract

The aim of this work is an alternative non destructive technique for estimating the thermal properties of four different Thermal Management System (TMS) materials. More in detail, a thermographic setup realized with the Active Thermography approach (AT) is utilized for the purpose and the data elaboration follows the ISO 18755 Standard.

As well known, Phase Changes Materials (PCMs) represent an innovative solution in the Thermal Management System (TMS) of Lithium-Ion batteries and, during the years, many solutions were developed to improve its thermal properties. As a matter of fact, parameters such as the internal temperature or heat exchanges impact on both efficiency and safety of the whole battery system. Consequently, the thermal conductivity was often chosen as a performance indicator of Thermal Management System (TMS) materials.

In this work, both thermal diffusivity and thermal conductivity were estimated in two different testing conditions, respectively at room temperature and higher temperature conditions. The Active Thermography (AT) technique proposed in this activity has satisfactory estimated both thermal diffusivity and thermal conductivity of Thermal Management System (TMS) materials.

An analytical model was also developed to reproduce the temperature experimental profiles. Finally, results obtained with AT approach were compared to those available from commercial datasheet and literature.

## Highlights

• Experimental thermal characterization of Thermal Management System materials.
• Fast and reliable Active Thermography approach based on ISO 18755 Standard.
• Thermal properties evaluation at different testing conditions.
• An analytical model to reproduce experimental results.
• Results compared to those available from material producer and literature.

## Nomenclature

d: Specimen thickness
h: Convection coefficient
k: Thermal conductivity
Q: Heat
$\dot{Q}$: Heat flux
$R_{conductive}$: Conductive resistance
$R_{eq}$: Equivalent thermal resistance
$R_{convective}$: Convective resistance
s: Sample surface
t_0.5_: Half rise time ISO18755
$T_{final}$: Temperature after the heating phase
$T_{start}$: Initial temperature
ΔT: Relative temperature
z: Thickness coordinate
α: Thermal diffusivity
γ: Coefficient for the model calibration
δ: Coefficient for geometry effects
η: Coefficient for the model calibration
φ: Coefficient for the model calibration
ρ: Density
σ: Coefficient for the material and environmental conditions

**Abbreviations**
AT: Active Thermography
EV: electric vehicle
GHG: Greenhouse gases
GHP: Guarded Hot Plate
HFM: Heat Flow Meter
LF: method and Laser Flash
LFPT: Laser Flash Pulsed technique
L-I: Lithium-ion
LIT: Lock-In technique
TMS: Thermal Management System
NDT: Non-Destructive Testing
PCM: Phase Change Material
PHEV: plug-in hybrids vehicle
PT: Passive Thermography
P-T: Pulsed Technique
ROI: Region Of Interest
TBC: Thermal Barrier Coatings

## 1 Introduction

Nowadays, vehicle electrification plays an important role in the ecological transition. Greenhouse gases (GHG) emissions caused by fossil powered transportation are decreasing with full electric and plug-in hybrids (EVs and PHEVs) vehicle structures. This way, carbon dioxide emissions reduction by using sustainable energy can be reached, achieving the goal of environmental benefit [1,2]. However, as illustrated in Refs. [3,4], high performance and comfort of vehicle may be guaranteed by using Lithium-ion (L-I) battery solutions which represents an affordable and convenient energy source, showing peculiarities of low cost, relatively high energy density and long duration [5].

A classical L-I battery configuration is made of several battery cells with arrangements of in series or in parallel structures. So, the required energy may be supplied and guaranteed to the whole system. On the other hand, the great heat generation release during the discharging phase may produce battery performance degradations or battery damages causing Runaway. To avoid this fail case, a controlled and monitored temperature between 20 and 40 °C is required to ensure system efficiency, as shown in Refs. [[6], [7], [8], [9]]. Anyhow, as suggested in Ref. [10], the complete knowledge of both thermal and physical properties and of management inside the batteries is relevant for high energy density and fast charging applications. To this aim a possible solution consists in Thermal Management System (TMS).

A TMS consists of a series of strategies adopted in L-I batteries to control their internal temperature to guarantee both efficiency and safety, managing heat dissipation and improving battery duration [7].

Several techniques were developed for TMS optimization, that is to decrease and control the temperature of the system. As an example, a passive strategy is performed by the so-called Phase Changes Material (PCM) method, well described in Ref. [11], where a PCM is infused in foam layers separating the L-I cells. Some additives or nanoparticles were also used to improve thermal properties of PCMs, as reported in Refs. [5,12,13].

More in detail, the PCM technique was widely utilized to enhance thermal properties, optimize heat exchanges and improve TMS characteristics, as described by many researchers [[13], [14], [15], [16], [17], [18], [19]].

Anyhow, the role of physical and thermal properties and their evaluation has become essential to improve both PCMs features and overall performance. As an example, an optimized thermal conductivity may allow a good temperature homogeneity in the phase change process, but a low thermal conductivity is needed between cells to prevent uncontrolled overheating. So, as an example [[20], [21], [22]], the thermal conductivity of cell separating material has to be deeply analysed to extend the cycle life of a power battery pack.

Conventional Non-Destructive Testing (NDT) methods for thermal diffusivity measurement of insulating materials include Heat Flow Meter (HFM) method, Guarded Hot Plate (GHP) method and Laser Flash (LF) method. In these methods, characterization of thermal diffusivity is referred to the well-known steady state or transient approaches. More in detail, GHP and HFM utilize a steady state analysis in which the thermal equilibrium of the investigated material is required for the material thermal diffusivity evaluation. On contrary, a transient condition is applied in case of Laser Flash (LF) method with the advantage of a very fast measurement of material thermal diffusivity. Anyhow, an accurate thermal diffusivity estimation is strictly related to both the sample size and the experimental setup. For this reason, some Standards, defining specimens characteristics, have to be considered when estimating thermal properties, such as ISO 8301 [23], ISO 8302 [24], ISO 22007-4 [25] and ISO/DIS 22482.2 [26]. This way, a more suitable thermal diffusivity characterization can be obtained.

During the years, many experimental Non-Destructive techniques, based on thermographic approach, were proposed for the thermal diffusivity characterization. For what concerns NDT, Thermography may represent a very promising solution in materials characterization field. Over the years, Passive Thermography (PT) was used to characterise fatigue damage in materials or components and to measure temperature distribution. In case of L-I battery applications [27], it was adopted to estimate the in-plane thermal conductivity of a flat rectangular sample by using both temperature distribution and heat transfer ratio. Experimental temperature measurements and distribution were used to calibrate the one dimensional conduction equation to obtain in plane thermal conductivity.

An alternative to the PT is the so-called stimulated thermography, also named Thermography in Active configuration or more simply Active Thermography (AT), which consist in analysing the thermal response of a surface to a heat input. As well known in literature, AT was applied to investigate hidden embedded in materials and components, and the scientific literature described methods and algorithms to improve defects visibility [[28], [29], [30], [31], [32]]. The main AT techniques are Lock-in technique (LIT), which investigates the phase and amplitude thermal response of a surface stimulated by a series of heat impulses and Pulsed Technique (P-T), which mainly investigates heating and cooling temperature profiles when a target surface is stimulated by a heat impulse. Laser Flash Pulsed technique (LFPT) consist in local focused monochromatic heat stimulation by means of a laser source. This technique is well described in ISO 18755 and ISO 18555, in particular for thermal diffusivity measurements in Thermal Barrier Coatings TBC and in multilayer coatings.

Other research fields were investigated. As an example, corrosion damage was studied in Refs. [33,34] considering a lack of material, and the phenomena was quantitatively characterised adopting a LIT. In case of material characterization from the thermal point of view, an AT setup was recently successfully implemented to estimate thermal diffusivities of Thermal Barrier Coatings (TBCs) [35,36] and Aerogel Materials [37]. In particular in Ref. [37] an alternative to the LFPT, according to ISO 18755 and ISO 18555 Standards [38,39], is proposed. A rapid and easier evaluation of both thermal diffusivity and thermal conductivity was performed without a dedicated equipment.

The present research activity aims at investigating the applicability of LFPT for assessing thermal diffusivity and thermal conductivity of cell separating materials used in TMS, to manage internal battery temperature and heat exchanges between parts.

In particular, four different TMS materials were thermally characterised with an Active Thermography (AT) setup and thermal parameters were evaluated according to ISO Standard [38].

Samples were adequately prepared to perform the thermal characterization. Thermal properties evaluation was performed at two testing conditions, at room temperature and higher temperature conditions.

A simple analytical model was also developed to predict thermal responses at different thermal excitations, and the influence of the model parameters was also investigated to reduce errors between experimental and simulated values. Results were compared with those available from commercial datasheet and literature.

## 2 Materials and methods

**Fig. 1:**
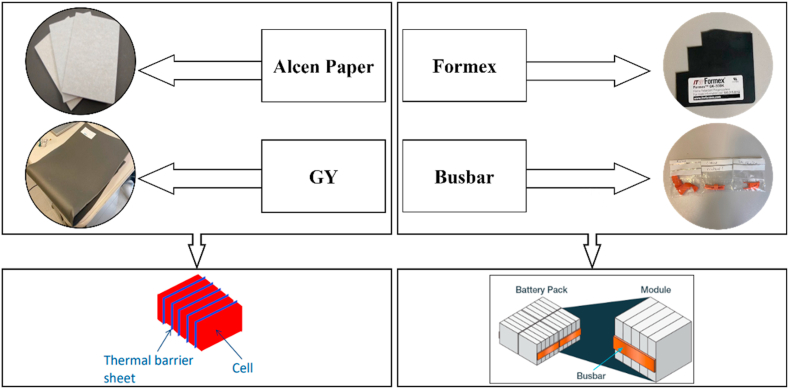
Thermal Management System (TMS) materials.

Four TMS materials (see Fig. 1) were analysed in this work: *Alcen Paper*, *GY*, *Formex* and PVC materials (made of three different components: *Busbar batteries*, *insulation sheath* and *insulating tape*). *Alcen Paper* and *GY* are commonly located between cells of battery modules, while *Formex* and PVC materials (*Busbar batteries*, *insulation sheath* and *insulating tape*) are adopted as a coating for the electronic part or as insulating materials in battery modules.

**Fig. 2:**
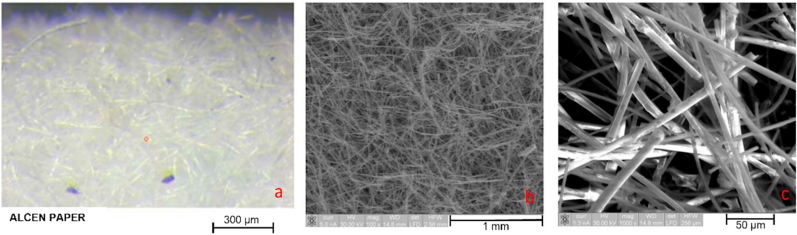
Optical (a) microscope and SEM (c, d) images for *Alcen Paper* sample (*t3*).

**Fig. 3:**
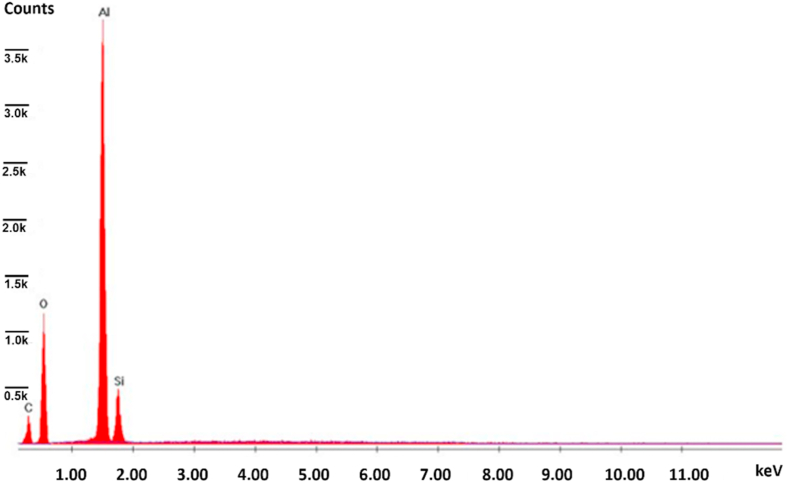
EDS analysis for *Alcen Paper* sample (*t3*).

**Fig. 4:**
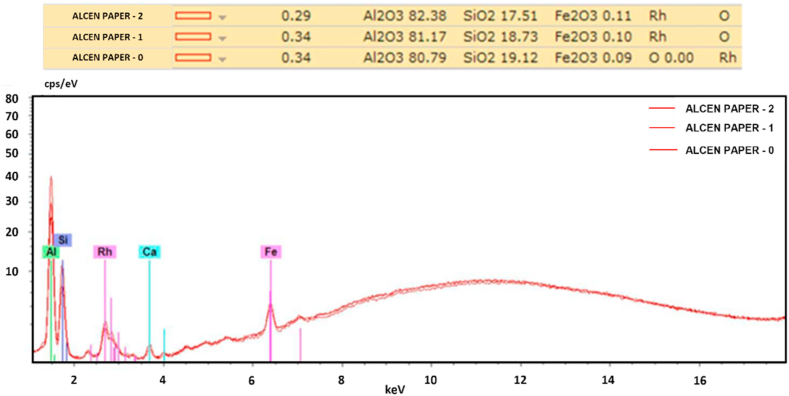
Quantitative analysis for *Alcen Paper* sample (*t3*).

**Fig. 5:**
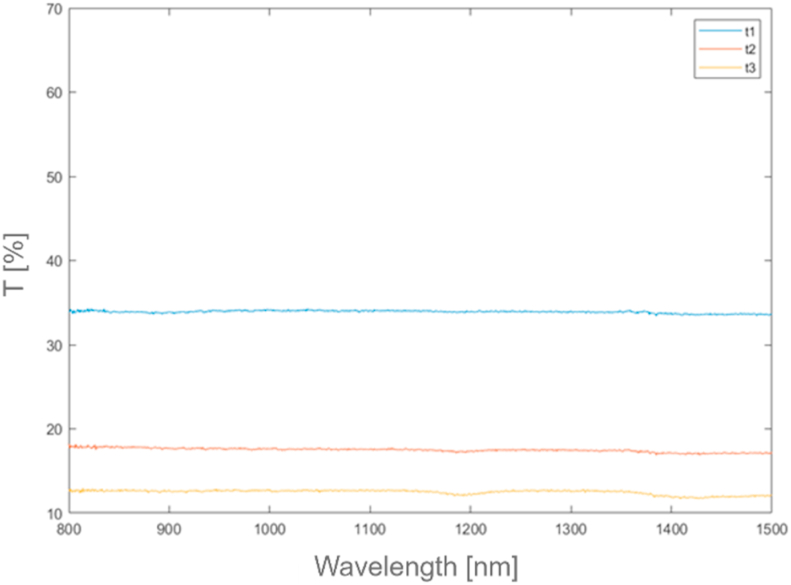
Transmittance spectrums for *Alcen Paper* samples (*t1, t2, t3*).

*Alcen Paper* showed a particular surface aspect, and it required further preliminary investigation before testing. Three different samples (*t1, t2, t3*) of *Alcen Paper* material were prepared by varying its thickness, respectively 1 mm (*t1*), 2 mm (*t2*), 3 mm (*t3*). Both SEM and EDS analysis and optical microscope acquisition were performed to analyse its microstructure and chemical composition. Some information about this preliminary investigation is reported in Fig. 2, Fig. 3, Fig. 4, Fig. 5. Fig. 2 shows optical microscope (a) and SEM (c, d) images for 3 mm *Alcen Paper* sample (*t3*). From the analysis of Fig. 2 (c, d) a fibrous material with a fibre diameter of about 3.8–12 μm (in some cases up to 30 μm) can be noted. EDS Spectrum (see Fig. 3) was detected with an acceleration voltage of 30 kV, showing an elementary composition of Al, O, Si and C. X ray spectrum photometer pointed out the corresponding quantitative composition (see Fig. 4) in three different zones (0, 1, 2) for sample *t3*, and results showed an alumina/silica ratio (Al2O3/SiO2) of about 80/20 with some Fe existence. Transmittance spectrum results for different wavelengths is reported in Fig. 5. It was obtained for each sample (*t1, t2, t3*) by means of spectrophotometer, according to ASTM E1348-15 [40]. From the analysis of Fig. 5, it can be noted that *t2* and *t3* samples were characterised by transmission values lower than sample *t1*, so *t2* and *t3* samples were selected for experiments due to their higher energy entity absorbed during the laser heating phase.

In general, thermal characterization requires samples with specific geometry to fulfil ISO 18755 Standard conditions [38]. A square geometry with an area of about 14 × 14 mm was chosen for all samples, while the corresponding thickness depended on the specific material. A small layer of black paint was deposited over *Alcen Paper* and PVC materials (*Busbar batteries*, *insulation sheath* and *insulating tape*) samples to improve its emissivity. Physical properties (specific heat and density) of each material were evaluated to compute the thermal conductivity (k) value from the corresponding experimental thermal diffusivity (α). The density was estimated after weight measurements (by a precision balance) and volume computation, while specific heat was found in material producer datasheets.

**Fig. 6:**
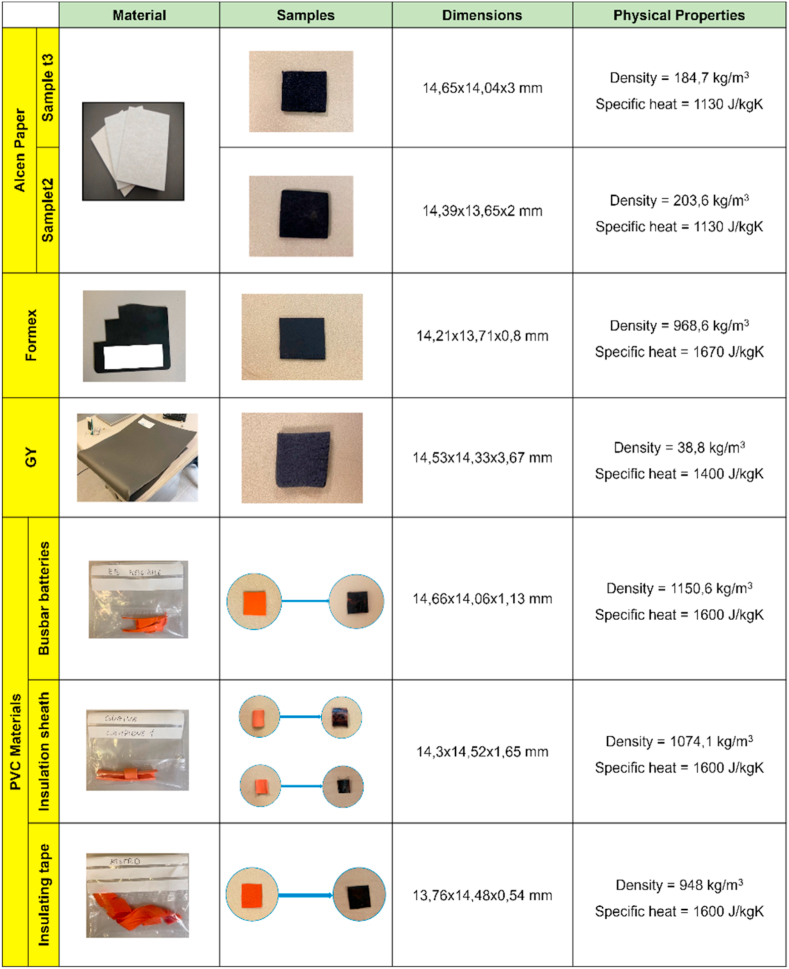
Samples dimensions and physical properties of all materials.

Samples dimensions and physical properties of all materials are summarised in Fig. 6.

A classical Active Thermography (AT) approach was used in this work for thermal characterisation.

AT equipment was composed of an IR thermo camera, a laser excitation source, and a PC control unit. The IR thermo camera was a FLIR X6540 with sensitivity lower than 20 mK and 3–5 μm spectral range, while the laser source could generate a maximum power of 30 W concentrated in a small surface (beam diameter about 6 mm with a Gaussian distribution). Thermal data were processed by using FLIR ResearchIR software and then results were elaborated with an in-house implemented Matlab routine to obtain local thermal profiles for the thermal diffusivity computation.

Thermal excitation is generally defined by power entity (% of the maximum power, %*P*_max_) and duration time (or time period). Specific thermal excitation ranges were tuned for each material to satisfy ISO 18755 Standard [38], and many excitation levels were covered for each sample to generate different energies heating.

Thermal properties were evaluated at two different testing conditions, room temperature and higher temperature. The room temperature condition corresponded to the classical environment temperature. The higher temperature testing condition corresponded to heat samples by a dedicated Muffle oven, where the surface temperature was controlled by a pyrometer. In particular, *Alcen Paper* and *GY* samples were heated respectively up to 90 °C and 50 °C, before thermography tests.

Laser power and duration time ranges utilized in each test, for both testing conditions (room temperature and in temperature conditions), and for each sample, are resumed in Table 1.

**Table 1:** Laser power and duration time.

Samples [−]		N° Test	Laser Power range [%*P*_max_ ]	Duration time range [ms]
**Alcen Paper**	**Sample t3**	6	50–60	10–20
	**Sample t2**	6	50–60	5–10
**Formex**		8	50–60	5–10
**GY**		6	50–60	5–10
**PVC materials**	**PVC Busbar batteries**	6	30–80	5
	**Insulation sheath**	6	40–80	5–10
	**Insulating tape**	6	11–60	5

**Fig. 7:**
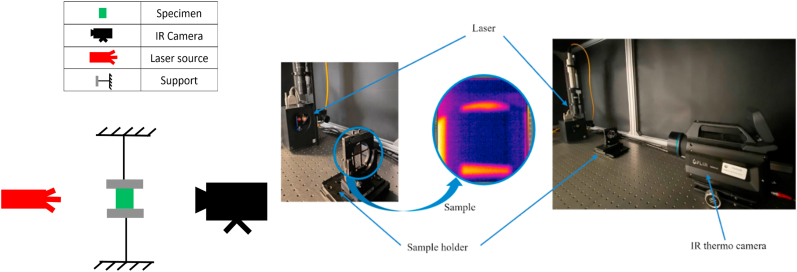
Experimental setup and all devices.

A transmission mode configuration was adopted for thermal characterization. A dedicated sample holder was designed to reduce heat dissipations during tests. Each specimen was clamped in the sample holder at the level of the laser source excitation and at 600 mm distance from the thermal camera. The frame rate acquisition was 477 Hz for each sample. Fig. 7 shows the experimental setup and all devices.

Thermal parameters (thermal diffusivity and thermal conductivity) were obtained basing on the method proposed in ISO 18755 Standard [38], by using the half rise time method starting from the relative temperature ΔT. More in detail, absolute temperature over time was acquired during Active Thermography tests and then a mean value was extracted referring to a Region Of Interest (ROI) located at the centre of the sample.

In all cases, the emissivity value was set to 0.9 based on the specimen black surface aspect.

Finally, the relative temperature ΔT (difference between the absolute temperature and the environmental temperature) was considered for the thermal diffusivity evaluation.

## 3 Analytical model

An analytical model was developed in Matalb environment to reproduce temperature profiles after the heating phase. This model was comprehensive of both convective and conductive heat transfer mechanisms, basing on the classical electrical analogy to model thermal phenomena [41].

Although thermal transient phenomena occurred during the Active Thermography tests, a steady state model was adopted due to the slow dynamics in the time domain.

**Fig. 8:**
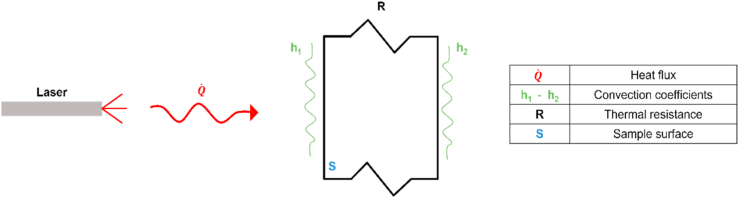
Equivalent thermal model.

Fig. 8 shows the equivalent thermal model, that describes by lumped parameters the heat flux $\dot{Q}$ provided by the laser source and its thermal interaction with the sample surface *S*.

The thermal excitation given by the laser source was considered as an energy flow, and the mathematical description was:

(1) $$\dot{Q}=\frac{\Delta T}{R_{eq}}=\frac{T_{final}-T_{start}}{R_{eq}}$$

where $\dot{Q}$ is the heat flux given by the laser source, ΔT the relative temperature ($T_{final}$, temperature reached after the heating phase, $T_{start}$, initial temperature) and $R_{eq}$ the equivalent thermal resistance. The equivalent thermal resistance $R_{eq}$ combined both convective effects ($R_{convective}\text{,}$ sample heat exchanges with the environment) and conductive effects ($R_{conductive}\text{,}$ heat transfer mechanisms inside the sample). More in detail:

(2) $$R_{convective}=\frac{1}{h}$$

(3) $$R_{conductive}=\frac{s}{k}$$

where h is the convection coefficient, s is the sample surface and k the thermal conductivity.

**Fig. 9:**
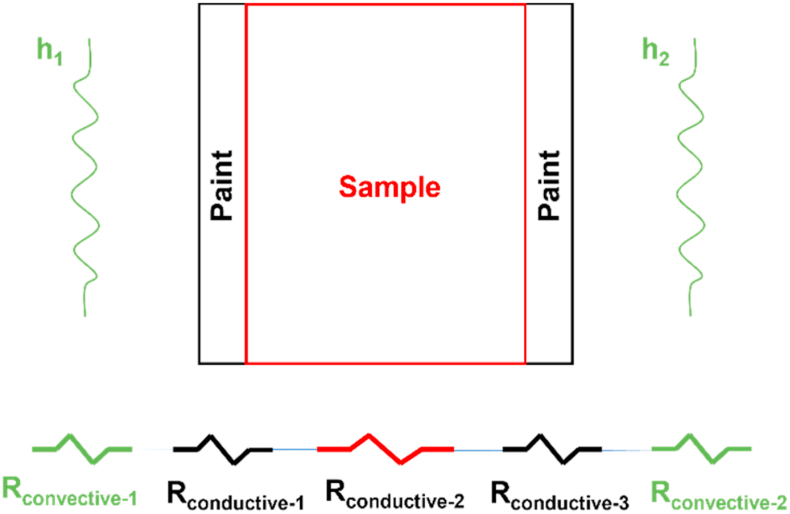
Equivalent thermal resistance.

In general, a sample can be black painted if necessary. This way, the heat flux phenomenon may be represented by: convection of the air gap, conduction of the black paint, conduction of the sample material. This thermal behaviour may be well described by the scheme of Fig. 9.

As illustrated in Fig. 9, the equivalent thermal resistance $R_{eq}$ may consider several heat flux phenomena on the basis of the analysed sample. As an example, the equivalent thermal resistance $R_{eq}$ for a sample with two black painted surfaces and convective phenomena (see Fig. 9) can be defined as:

(4) $$R_{eq}=R_{convective-1}+R_{conductive-1}+R_{conductive-2}+R_{conductive-3}+R_{convective-2}==\frac{1}{h_{1}}+\frac{s_{1}}{k_{1}}+\frac{s_{2}}{k_{2}}+\frac{s_{3}}{k_{3}}+\frac{1}{h_{2}}$$

where:

• $R_{convective-1}$ = $R_{convective-2}$ ($h_{1}$ = $h_{2}$): convective thermal resistance due to convective heat exchanges between black painted surfaces (black coating) and the air,
• $R_{conductive-1}$: conductive thermal resistance of the heated black painted surface,
• $R_{conductive-2}$: conductive thermal resistance of the sample,
• $R_{conductive-3}$: conductive thermal resistance of the monitored black painted surface.

Once calculated the equivalent thermal resistance $\left(R_{eq}\right)$, the temperature reached after the heating phase ($T_{final}$) can be obtained by rearranging Equation (1) in the following:

(5) $$T_{final}=T_{start}+\dot{Q∙}R_{eq}$$

where an appropriate mathematical function for the heat flux ($\dot{Q}$) was chosen to reproduce the so-called “in transmission mode” temperature profile.

The classical mono-dimensional analytical formulation in the transient regime [31] may be represented by the following relationship:

(6) $$T_{final\left(z,t\right)}=T_{start}+\frac{Q}{\sqrt{k\rho c_{p}t}}e^{\left(-\frac{z^{2}}{4\alpha t}\right)}$$

where z is the general coordinate along the body's thickness, ρ is the density, α is the thermal diffusivity and Q is the heat. Rearranging Equations (5), (6), the appropriate heat flux formulation ($\dot{Q}$) can be defined as follows.

(7) $$T_{start}+\dot{Q}∙R_{eq}=T_{start}+\frac{Q}{\sqrt{k\rho c_{p}t}}e^{\left(-\frac{z^{2}}{4\alpha t}\right)}$$

So, from Equation (8) the final formulation for the heat flux ($\dot{Q}$) in case of a stationary description can be obtained:

(8) $$\dot{Q}=\frac{Q}{\sqrt{k\rho c_{p}t}}e^{\left(-\frac{d^{2}}{4\alpha t}\right)}∙\frac{1}{R_{eq}}$$

where in this case d is the specimen thickness. By integrating Equation (8), and considering the exponential contribution as a constant term due to the slow dynamics in the time domain, the final formulation of the heat Q is defined as follows:

(9) $$Q\left(t\right)={\varphi ∙e}^{\frac{2∙\sqrt[{2∙\gamma }]{t}}{{\sigma ∙e}^{\frac{\eta ∙\delta }{t}}}}$$

and several parameters were defined during the integration. In detail:

(10) $$\delta =\frac{d^{2}}{4∙\alpha }$$

(11) $$\sigma =\sqrt{\varepsilon }∙R_{eq}=\sqrt{k∙\rho ∙c_{p}∙\pi }∙R_{eq}$$

where:

• δ considers only the sample dimension (thickness), and it is calibrated for each tested material (see Equation (10)),
• σ is related to material and environmental properties (see Equation (11)).

**Fig. 10:**
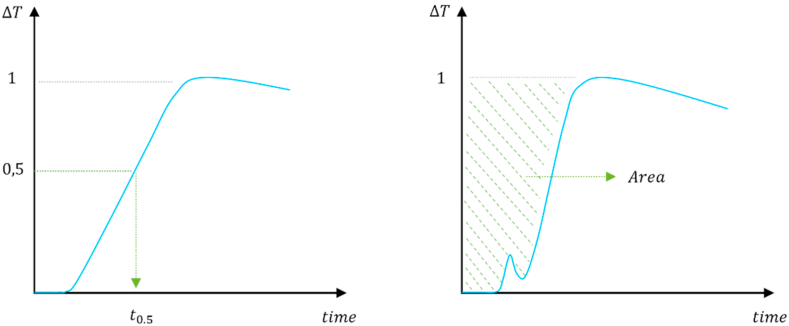
Temperature profiles (ΔT) illustrated in ISO Standards [38,39].

The other coefficients (φ, η and γ) were introduced in the final formulation (Equation (9)) to better calibrate the model. In case of regular temperature profiles (see Fig. 10 on the left), the coefficients tuning is based on a superimposition between experimental and analytical curves. If the temperature profile shows an initial noise (see Fig. 10 on the right), the calibration phase requires a different approach. More in detail, it was realized by varying coefficients until the experimental and the analytical thermal area (ISO 18555 [39]) reached the same value.

Additional coefficients are φ,η,γ:

• φ depends on the laser power and it influences the reconstructed profile with a vertical variation,
• η is related to both time period and sample thickness and it influences both the pick value and the heating slope,
• γ affects only the cooling phase. This parameter was set equal to one since it isn't relevant in the thermal diffusivity evaluation.

**Fig. 11:**
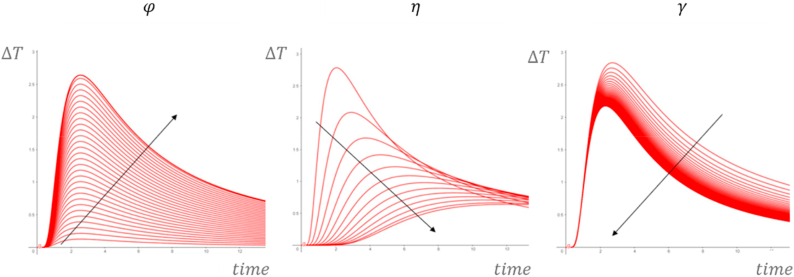
Influence of φ, η and γ.

Fig. 11 shows the influence of φ, η and γ on the relative temperature profile.

In detail, the model solves the thermal transmission equation in space and time in a multilayer flat infinite surface (equation (6)), given the heat input distribution in time (equation (9)). The materials properties consist in the calibration parameters for equation (9).

In initial condition the whole multilayer plate is at room temperature and at boundary condition the room temperature is constant.

In conclusion, the temperature reached at the end of the heating phase can be reproduced by using a stationary description (Equation (5)) with an appropriate mathematical formulation (Equation (9)). This way, the thermal diffusivity may be computed from the reproduced heating profile, which is a function of both material properties and laser thermal excitations (laser power and duration time).

The analytical model was validated for two materials: Alcen paper and GY. The corresponding physical material properties, required to calibrate the models, are reported in Fig. 6. The thermal properties used in the model are the ones obtained experimentally and reported in the following Results section.

## 4 Results

In this section, results regarding the thermal characterization are shown. In the initial part experimental results are illustrated, while in the last one the analytical modelling is presented.

Thermal conductivity of each sample (*Alcen Paper*, *GY*, *Formex, Busbar batteries*, *insulation sheath* and *insulating tape*) was evaluated from measurement at room temperature condition.

*Alcen Paper* and *GY* samples were also characterized at higher temperature condition.

**Fig. 12:**
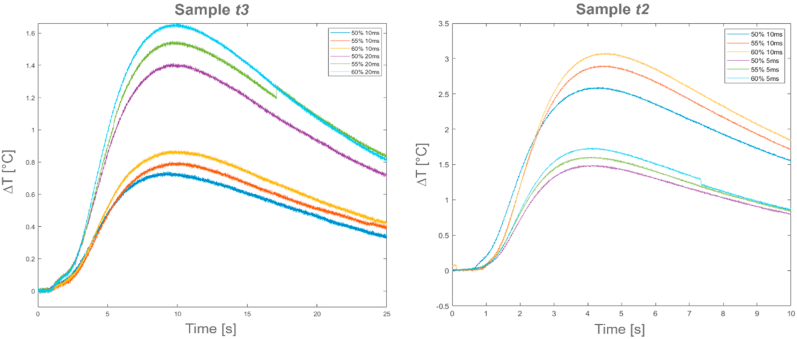
Relative temperature profiles (ΔT) for *t3* and *t2 Alcen Paper* samples.

Fig. 12 illustrates the relative temperature profiles (ΔT) for the *Alcen paper* material at different thermal excitations (left side for sample *t3* and right side for sample *t2*) and at room temperature. As it can be observed from Fig. 12, an initial trigger was necessary to start the recording before the laser excitation.

Table 1, Table 2, Table 3, Table 4, Table 5 show the experimental results for *Alcen Paper* material (*t3* samples*,* Table 2 and 4*,* and *t2* samples, Table 3, Table 5), respectively at room temperature (Table 2, Table 3) and higher temperature (Table 4, Table 5). In particular, all tables show: test number, power laser entity, duration time, half rime time (*t*_*0.5*_), thermal diffusivity (α) and thermal conductivity (k). All Standard conditions referred to the half rime time (*t*_*0.5*_) were satisfied [38].

**Table 2:** Sample *t3* (*Alcen Paper*) results at room temperature condition.

Test [−]	Power laser [%]	Duration time [ms]	t_0.5_ [s]	α [m^2^/s]	k [W/mK]
**1** **2** **3** **4** **5** **6**	50 55 60 50 55 60	20 20 20 10 10 10	3,63 3,66 3,64 3,51 3,52 3,65	3,44E-07 3,41E-07 3,43E-07 3,55E-07 3,54E-07 3,42E-07	0,0718 0,0713 0,0716 0,0743 0,0741 0,0714

**Table 3:** Sample *t2* (*Alcen Paper*) results at room temperature condition.

Test [−]	Power laser [%]	Duration time [ms]	t_0.5_ [s]	α [m^2^/s]	k [W/mK]
**1** **2** **3** **4** **5** **6**	50 55 60 50 55 60	10 10 10 5 5 5	1,79 1,69 1,77 1,56 1,62 1,69	3,10E-07 3,29E-07 3,14E-07 3,56E-07 3,43E-07 3,29E-07	0,0758 0,0803 0,0796 0,0870 0,0839 0,0805

**Table 4:** Sample *t3* (*Alcen Paper*) results at higher temperature condition.

Test [−]	Power laser [%]	Duration time [ms]	t_0.5_ [s]	α [m^2^/s]	k [W/mK]
**1** **2** **3** **4** **5** **6** **7** **8**	50 50 50 50 50 50 50 50	10 10 10 10 10 10 10 10	1,01 1,70 1,34 1,83 2,34 2,02 2,46 3,28	1,24E-06 7,32E-07 9,33E-07 6,82E-07 5,33E-07 6,18E-07 5,07E-07 3,81E-07	0,2491 0,1476 0,1878 0,1375 0,1075 0,1246 0,1024 0,0769

**Table 5:** Sample *t2* (*Alcen Paper*) results at higher temperature condition.

Test [−]	Power laser [%]	Duration time [ms]	t_0.5_ [s]	α [m^2^/s]	k [W/mK]
**1** **2** **3** **4** **5** **6** **7** **8**	50 50 50 50 50 50 50 50	10 10 10 10 10 10 10 10	1,08 1,02 1,14 1,02 1,10 1,43 1,43 1,52	5,14E-07 5,44E-07 4,87E-07 5,44E-07 5,04E-07 3,88E-07 3,72E-07 3,65E-07	0,1258 0,1332 0,1192 0,1333 0,1237 0,0953 0,0915 0,0898

Results for the other tested materials (*GY*, *Formex, Busbar batteries*, *insulation sheath* and *insulating tape*) are organized in a similar way.

**Fig. 13:**
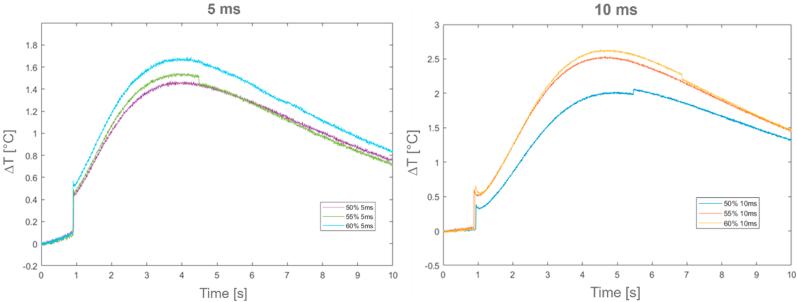
*GY* relative temperature profiles (ΔT) at 5 ms and 10 ms time period excitations.

Fig. 13 reports the relative temperature profiles (ΔT) in case of room temperature conditions for the *GY* sample (on the left for a duration time of 5 ms, on the right for a duration time of 10 ms).

Table 6, Table 7 report thermal characterization results and related excitation parameters for the *GY* sample, in both room and higher temperature conditions. Only results up to a laser excitation power of 60% are presented, as over this power value a sample degradation occurred.

**Table 6:** *GY* results at room temperature condition.

Test [−]	Power laser [%]	Duration time [ms]	*t*_*0.5*_ [s]	α [m^2^/s]	k [W/mK]
1 2 3 4 5 6	50 55 60 50 55 60	10 10 10 5 5 5	1,90 1,74 1,75 1,24 1,23 1,20	3,10E-07 3,29E-07 3,14E-07 3,56E-07 3,43E-07 3,29E-07	0,0758 0,0803 0,0796 0,0870 0,0839 0,0805

**Table 7:** *GY* results during at higher condition.

Test [−]	Power laser [%]	Duration time [ms]	*t*_*0.5*_ [s]	α [m^2^/s]	k [W/mK]
1 2 3 4 5 6 7 8	50 50 50 50 50 50 50 50	5 5 5 5 5 5 5 5	0,81 0,75 0,94 1,14 1,10 2,37 1,13 1,20	2,30E-06 2,49E-06 1,98E-06 1,64E-06 1,69E-06 7,88E-06 1,65E-06 1,55E-06	0,126 0,136 0,108 0,089 0,092 0,043 0,090 0,085

Also in these cases, all Standard conditions referred to half rime time (*t*_*0.5*_) were satisfied [38].

**Fig. 14:**
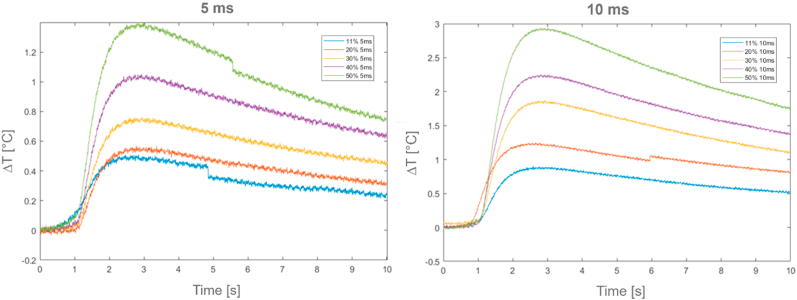
*Formex* relative temperature profiles (ΔT) at 5 ms and 10 ms time period excitations.

Fig. 14 and Table 8 report the thermal characterization values for *Formex* sample, at room temperature. Also in these cases, all Standard conditions referred to half rime time (*t*_*0.5*_) were satisfied [38]. In the same figure the apparent “step” present in some cooling profiles is related to missing frames in thermographic acquisition, due to the high acquisition frequency rate. As the cooling phase is not relevant in thermal diffusivity computation, this irregularity was not compensated.

**Table 8:** *Formex* results.

Test [−]	Power laser [%]	Duration time [ms]	*t*_*0.5*_ [s]	α [m^2^/s]	k [W/mK]
**1** **2** **3** **4** **5** **6** **7** **8** **9** **10**	11 20 30 40 50 11 20 30 40 50	10 10 10 10 10 5 5 5 5 5	1,08 1,04 1,16 1,04 1,13 1,08 1,08 0,96 1,06 1,12	8,22E-08 8,54E-08 7,66E-08 8,54E-08 7,86E-08 8,22E-08 8,22E-08 9,25E-08 8,38E-08 7,93E-08	0,133 0,138 0,124 0,138 0,127 0,133 0,133 0,150 0,136 0,128

The thermal characterization results for PVC materials are organized referring to each single components, as *Busbar batteries, insulation sheath and insulating tape* were tested separately, only at room temperature.

**Fig. 15:**
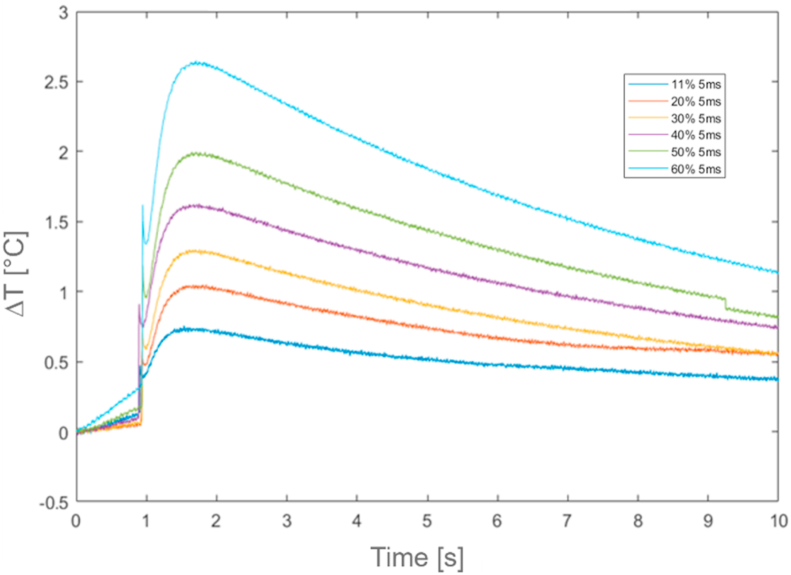
Relative temperature profiles (ΔT) for *Busbar batteries*.

**Fig. 16:**
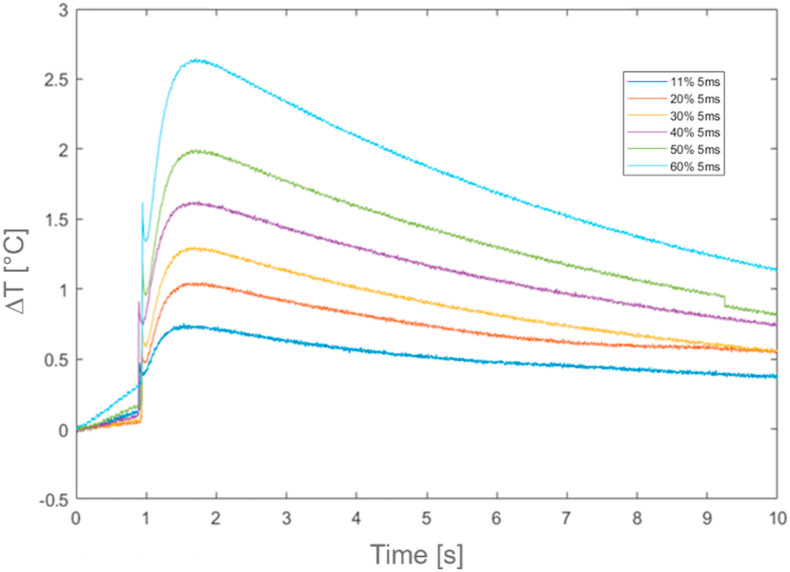
Relative temperature profiles (ΔT) for *insulating tape*.

**Fig. 17:**
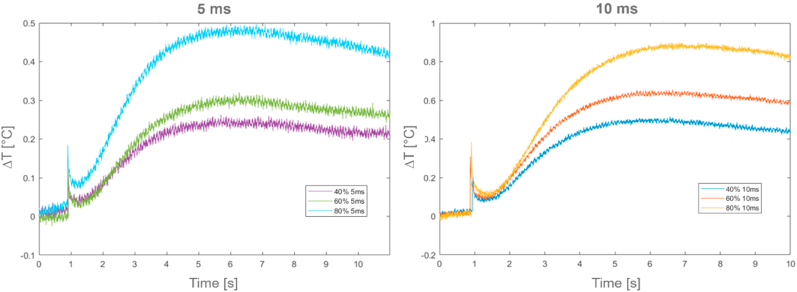
Relative temperature profiles (ΔT) for *insulating sheath* at 5 ms and 10 ms time period excitation.

Fig. 15, Fig. 16, Fig. 17 and Table 9, Table 10, Table 11 report all results of the thermal characterization for PVC materials components. For these materials, thermal responses are characterized by a typical phenomenon, known as Initial Noise, indicated by the sudden initial spike in temperature, well described in ISO 18755 Standard.

**Table 9:** *Busbar batteries* results.

Test [−]	Power laser [%]	Duration time [ms]	*t*_*0.5*_ [s]	α [m^2^/s]	k [W/mK]
**1** **2** **3** **4** **5** **6**	30 40 50 60 70 80	5 5 5 5 5 5	1,45 1,42 1,44 1,45 1,47 1,45	1,22E-07 1,22E-07 1,22E-07 1,22E-07 1,22E-07 1,22E-07	0,255 0,230 0,227 0,225 0,222 0,225

**Table 10:** *Insulating tape* results.

Test [−]	Power laser [%]	Duration time [ms]	*t*_*0.5*_ [s]	α [m^2^/s]	k [W/mK]
**1** **2** **3** **4** **5** **6**	11 20 30 40 50 60	5 5 5 5 5 5	0,75 0,75 0,78 0,76 0,78 0,79	6,04E-08 6,23E-08 5,70E-08 6,04E-08 6,13E-08 5,47E-08	0,092 0,094 0,086 0,092 0,093 0,083

**Table 11:** *Insulating sheath* results.

Test [−]	Power laser [%]	Duration time [ms]	*t*_*0.5*_ [s]	α [m^2^/s]	k [W/mK]
**1** **2** **3** **4** **5** **6**	40 60 80 40 60 80	10 10 10 5 5 5	2,12 2,19 2,41 2,04 2,28 2,11	1,78E-07 1,73E-07 1,57E-07 1,85E-07 1,66E-07 1,79E-07	0,306 0,297 0,269 0,318 0,285 0,308

**Fig. 18:**
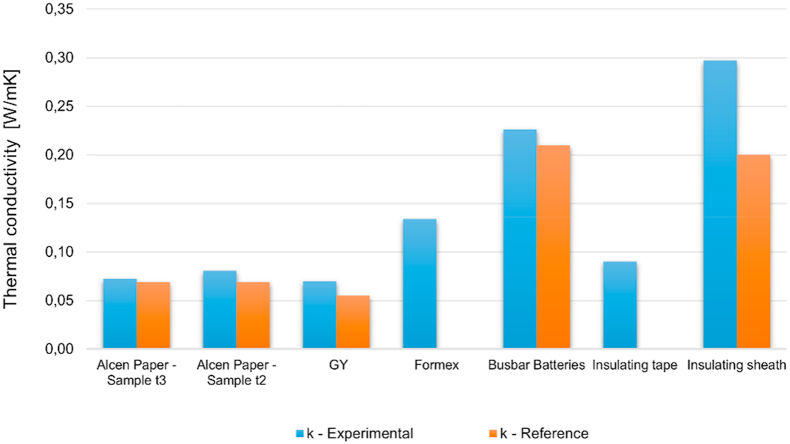
Thermal conductivities comparison at room temperature condition.

Fig. 18 illustrates the averaged thermal conductivity values obtained from the experimental characterization at room temperature. An experimental uncertainty of around 0.007 W/mK was estimated from the standard deviation of all experimental tests. Results were compared, where available, with those provided by producers (*Alcen Paper* and *insulating sheath*) or by literature (*GY* [42], *Busbar battery* [43]).

**Fig. 19:**
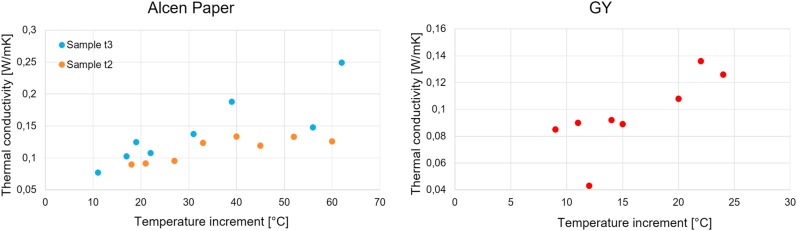
Higher temperature condition results.

Fig. 19 shows some preliminary results obtained for *Alcen Paper* and *GY* materials in higher temperature tests. In particular, Fig. 19 points out the increment in thermal conductivity values by increasing the sample surface temperature (reached after the heating phase in the oven).

In the following, analytical results and the comparison with experimental ones are illustrated. Firstly, the best tuning between experimental and numerical temperature profiles was investigated for *Alcen Paper* and *GY* materials, and related coefficients values can be seen in Table 12, Table 13, Table 14.

**Table 12:** *Alcen Paper* Sample *t3* coefficients.

Test [−]	Laser power [%]	Duration time [ms]	σ [s^1/2^]	δ [s]	φ [W/m^2^]	η [−]	γ [−]
**1** **2** **3** **4** **5** **6**	50 55 60 50 55 60	20 20 20 10 10 10	150 150 150 150 150 150	6,7 6,7 6,7 6,7 6,7 6,7	2150 2300 2500 1060 1130 1250	2,6 2,6 2,6 2,45 2,45 2,45	1 1 1 1 1 1

**Table 13:** *Alcen Paper* Sample *t2* coefficients.

Test [−]	Laser power [%]	Duration time [ms]	σ [s^1/2^]	δ [s]	φ [W/m^2^]	η [−]	γ [−]
**1** **2** **3** **4** **5** **6**	50 55 60 50 55 60	10 10 10 5 5 5	173,5 173,5 173,5 173,5 173,5 173,5	2,98 2,98 2,98 2,98 2,98 2,98	980 1110 1180 550 600 640	2,12 2,12 2,12 2 2 2	1 1 1 1 1 1

**Table 14:** *GY* coefficients.

Test [−]	Laser power [%]	Duration time [ms]	σ [s^1/2^]	δ [s]	φ [W/m^2^]	η [−]	γ [−]
**1** **2** **3** **4** **5** **6**	50 55 60 50 55 60	10 10 10 5 5 5	76,6 76,6 76,6 76,6 76,6 76,6	2,63 2,63 2,63 2,63 2,63 2,63	340 450 470 210 220 240	2,52 2,52 2,52 1,8 1,8 1,8	1 1 1 1 1 1

**Fig. 20:**
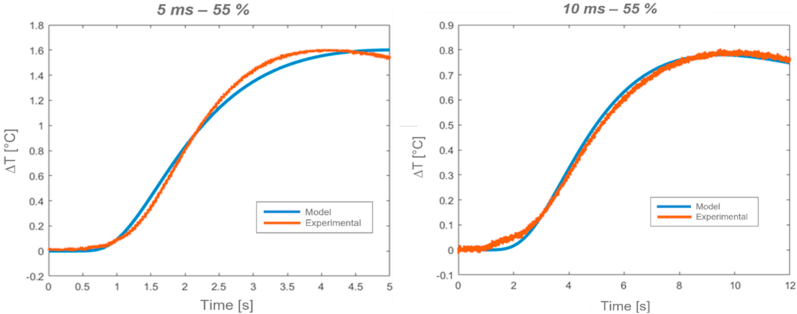
*Alcen Paper* (Sample *t2*, on the left, Sample *t3*, on the right).

**Fig. 21:**
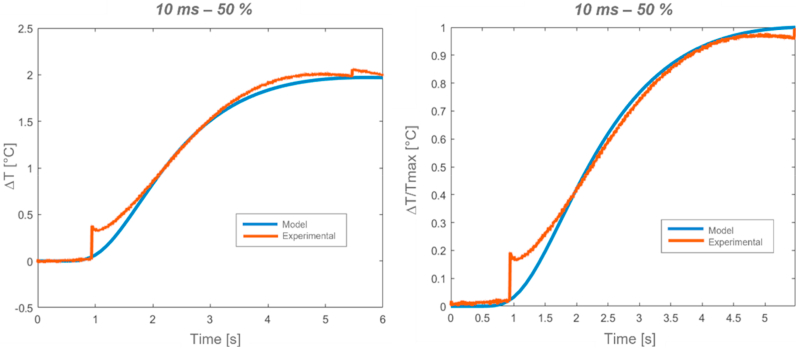
*GY* (relative temperature, left, normalized temperature, right).

An example of computed relative temperature profiles (ΔT), and the corresponding normalized profiles, for two different thermal excitations is respectively illustrated in Fig. 20, Fig. 21. Matlab software was utilized for the computation.

From the analysis of Fig. 20, Fig. 21, it can be pointed out that good approximation between experimental and analytical results was achieved. In particular, both half rise time intersection and corresponding thermal diffusivity values were guaranteed.

## 5 Conclusions

Aim of the present research activity is to investigate the applicability of Non Destructive AT for assessing thermal diffusivity and thermal conductivity of cell separating materials for Thermal Management System materials. This solution was also to be intended as an alternative to the ISO 18755 Standard.

The results obtained in the present work allowed to draw the following conclusions.

The AT technique proposed in this activity has satisfactory estimated both thermal diffusivity and thermal conductivity of cell separating materials for Thermal Management System (TMS) materials if compared with literature data and material producer datasheets.

Particular attention was required during samples preparation to fulfil ISO Standard recommendations.

The two testing conditions utilized, room temperature and higher temperature, showed the possibility to apply AT approach in several practical applications.

The developed analytical model, despite the simplifying assumptions, provided satisfying results for all analysed materials in terms of temperature profiles. Half rise time or areal heat diffusion time requirements (ISO 18755 and 18555 Standards) were always guaranteed by the analytical model in order to estimate thermal diffusivity values.

Furthermore, possible coefficient correlations were also investigated to reproduce relative temperature profiles at different laser thermal excitations.

In conclusion, all results were comparable and in a good agreement with those proposed by the material manufacturers datasheet or available in literature.

## CRediT authorship contribution statement

**Francesca Curà:** Supervision, Methodology, Conceptualization. **Raffaella Sesana:** Writing – review & editing, Validation, Data curation, Conceptualization. **Luca Corsaro:** Writing – original draft, Methodology, Conceptualization. **Marie Marguerite Dugand:** Supervision, Resources, Methodology.

## Declaration of competing interest

The authors declare that they have no known competing financial interests or personal relationships that could have appeared to influence the work reported in this paper.
